# Supplementary material for: Visualizing the dynamics of exported bacterial proteins with the chemogenetic fluorescent reporter FAST
Source: Sci Rep. 2020 Sep 25;10:15791. doi: 10.1038/s41598-020-72498-2 (PMC7519654; doi:10.1038/s41598-020-72498-2)
Supplement: Supplementary file 1 — Supplementary information. [file 41598_2020_72498_MOESM1_ESM.pdf]

SUPPLEMENTARY INFORMATION

Visualizing the dynamics of exported bacterial proteins with the chemogenetic fluorescent reporter FAST

Yankel Chekli[1,2], Caroline Peron-Cane[3,4], Dario Dell'Arciprete[3], Jean-François Allemand[3,4], Chenge Li[5], Jean-Marc Ghigo[1], Arnaud Gautier[5,6,7], Alice Lebreton[4,8], Nicolas Desprat[3,4,*] and Christophe Beloin[1,*]

[1] Genetics of Biofilms Laboratory, Institut Pasteur, UMR CNRS2001, Paris, 75015, France.

[2] Université de Paris, Sorbonne Paris Cité, Paris, France.

[3] Laboratoire de Physique de l'ENS, École Normale Supérieure, Université PSL, CNRS, Sorbonne Université, Université de Paris, 75005 Paris, France.

[4] Institut de biologie de l'ENS (IBENS), École Normale Supérieure, CNRS, INSERM, Université PSL, 75005 Paris, France.

[5] Sorbonne Université, École Normale Supérieure, Université PSL, CNRS, Laboratoire des Biomolécules (LBM), Paris, 75005, France.

[6] PASTEUR, Department of Chemistry, École Normale Supérieure, PSL University, Sorbonne Université, CNRS, 75005 Paris, France.

[7] Institut Universitaire de France.

[8] INRAE, IBENS, 75005 Paris, France.

Supplementary Figures

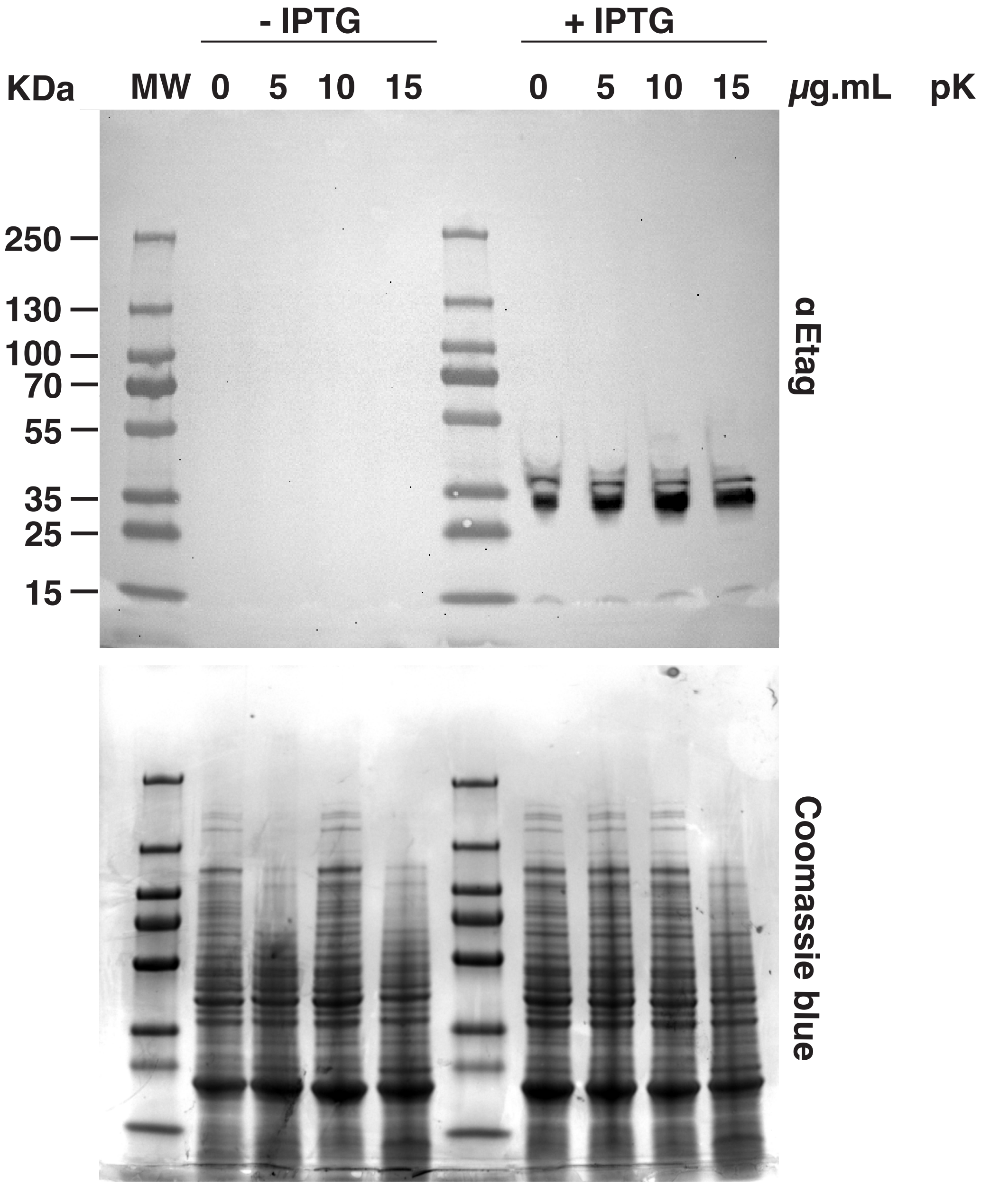

Figure S1. The Pf3-FAST chimeric cytoplasmic protein is not degraded by proteinase K. *E. coli* MG1655 harbouring pPf3-FAST (expressing Pf3-FAST construct) cells were grown overnight in absence or presence of 0.25 mM IPTG to induce the production of the chimeric Pf3-FAST protein. Bacteria were treated with 0, 5, 10 or 15 µg.mL^{-1} of proteinase K for 15 minutes at 40°C before western blot of whole-cell protein extracts and revelation with a rabbit anti-E-tag primary antibody and a HRP-linked anti-rabbit secondary antibody. The corresponding coomassie blue gel of the same samples is presented below as a control of equivalent samples loading.

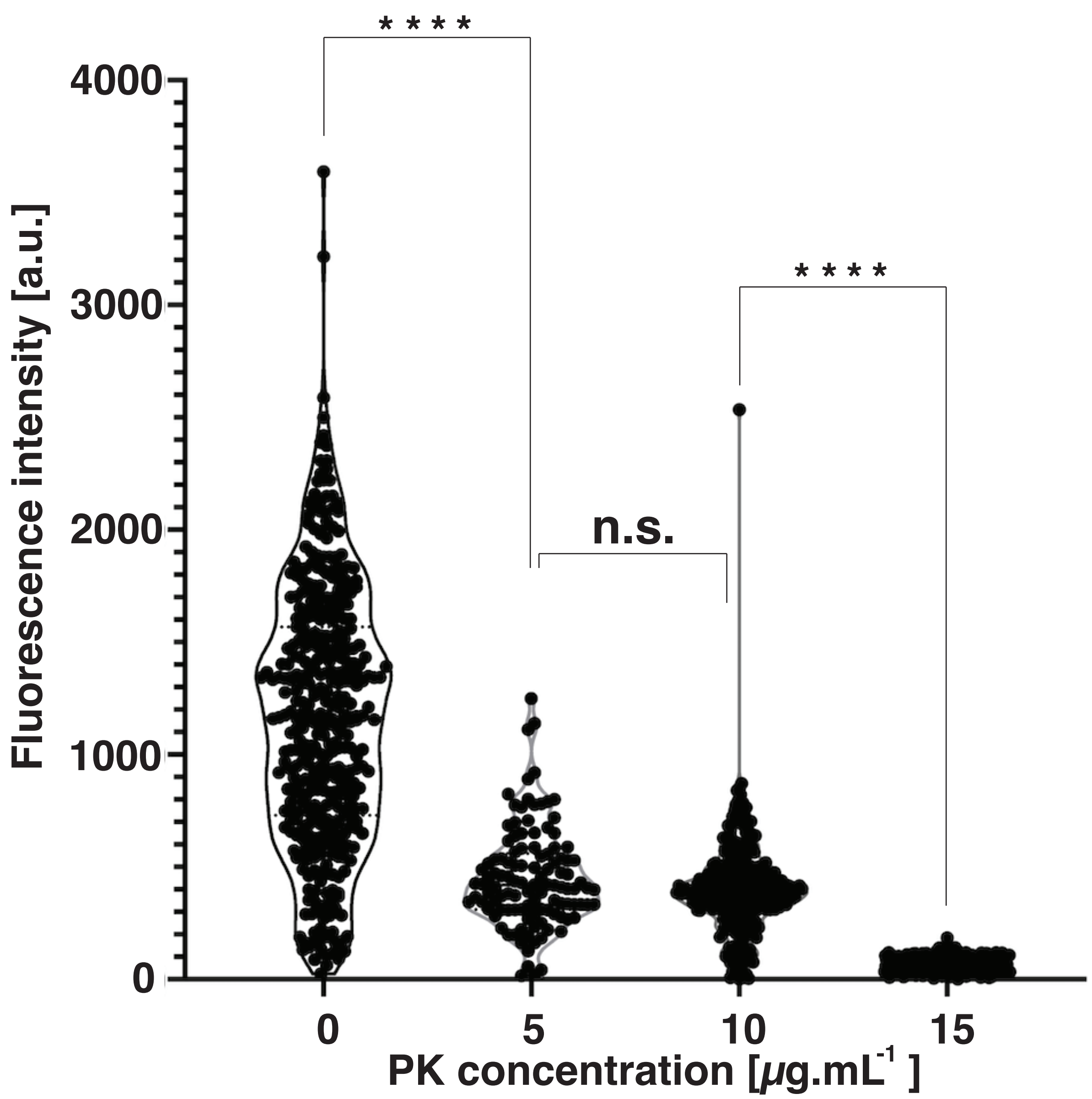

Figure S2. Proteinase K degrades surface exposed FAST. Quantification of fluorescence intensity from immunofluorescence images generated in Figure 2B. The distributions of fluorescence intensities are represented through violin plots for four values of proteinase K (PK) concentration. Data point sample size: n = (188, 143, 192, 78) for (0, 5, 10, 15) µg.mL^{-1} proteinase K concentration.

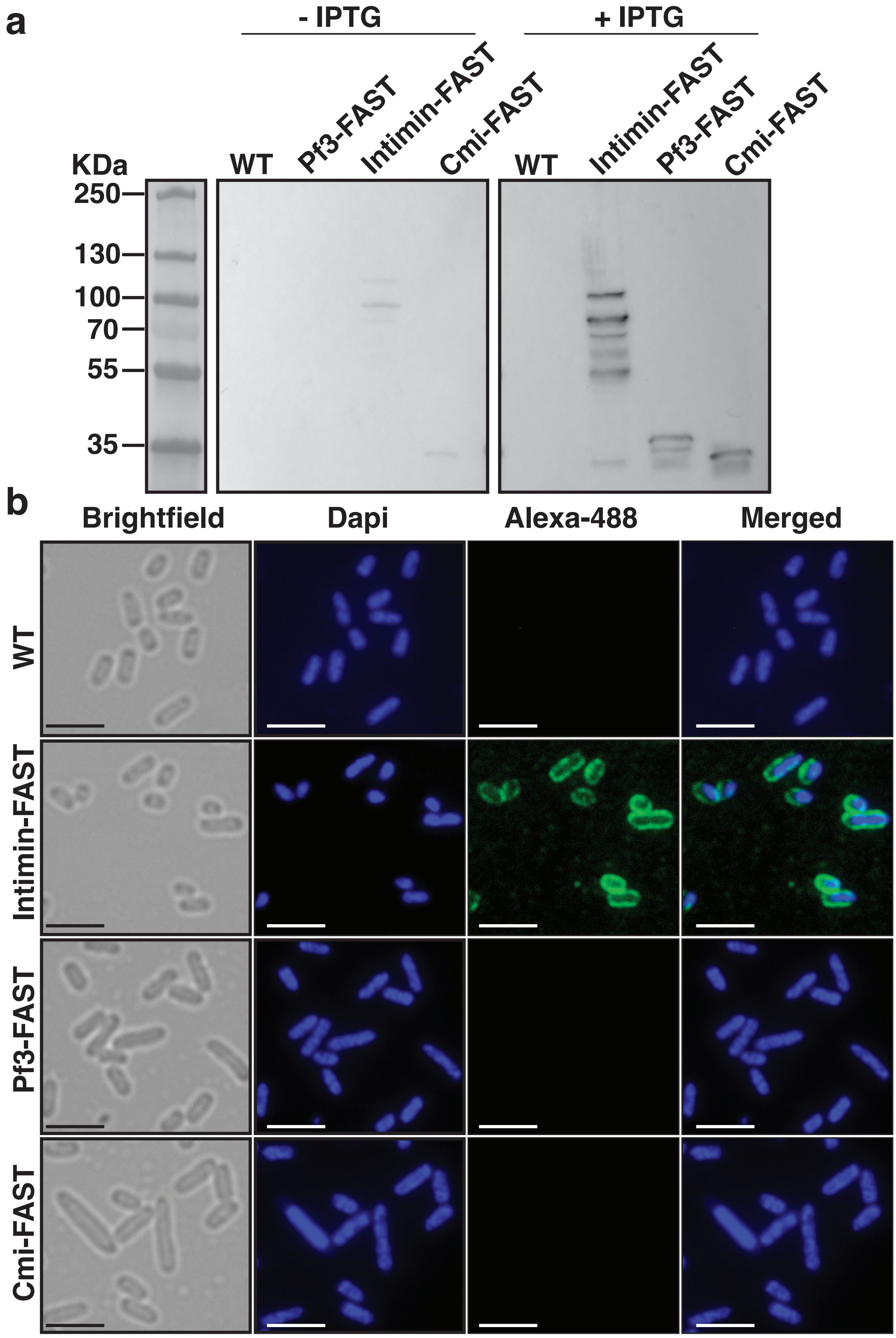

Figure S3. Pf3 and Cmi-FAST fusions are produced by bacteria and are not detectable by immunofluorescence confirming their cytoplasmic/periplasmic localization. a : Western blot of whole-cell protein extracts from *E. coli* MG1655, either uninduced (left pannel) or induced with 0.25 mM IPTG until OD 1 (right pannel), harbouring either no plasmid, pNeae2-FAST, pPf3-FAST or pCmi-FAST. The image has been cropped from a larger blot as depicted in Supplementary Information file S8. **b:** Immunofluorescence performed on the corresponding cells, without permeabilisation. The bacterial surface was labeled with an anti-E-tag primary antibody and an anti-rabbit Alexa 488 conjugated secondary antibody. Scale bars = 2 µm.

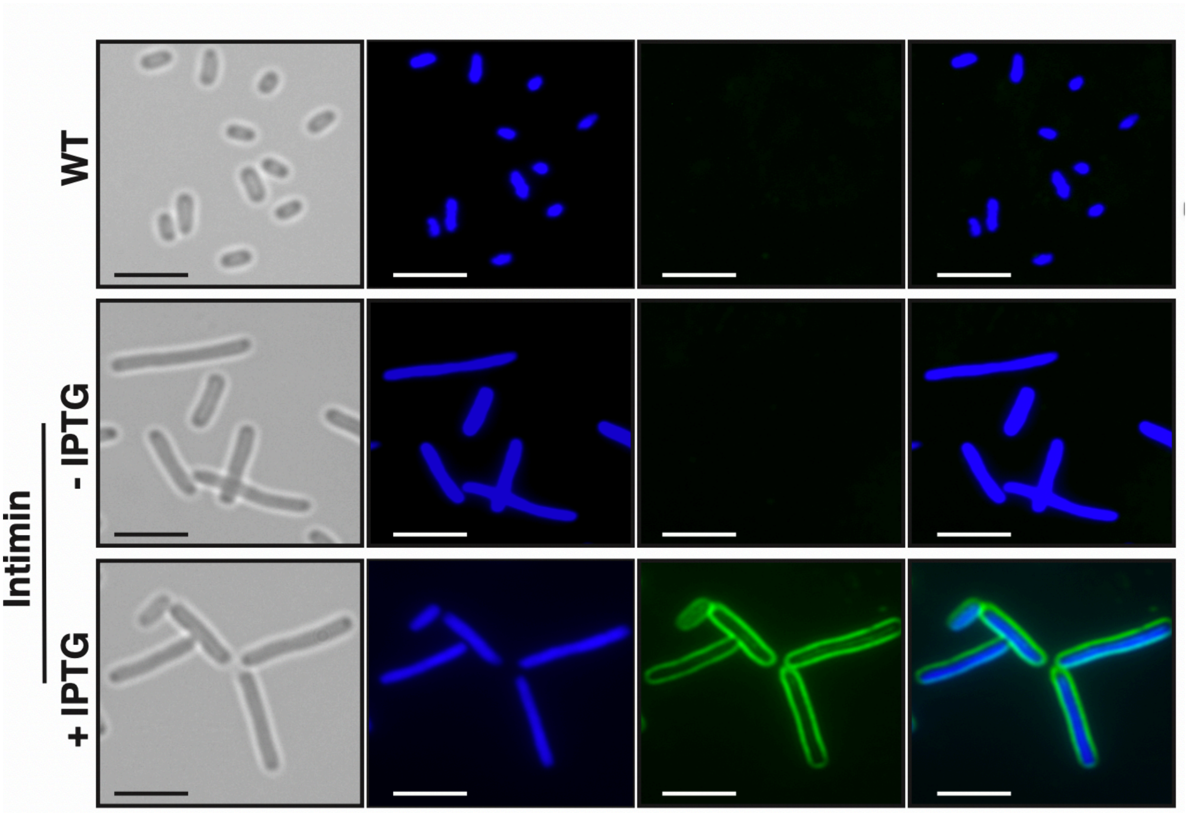

Figure S4. Presence of the pNeae2 plasmid itself induce an elongation of the cells regardless of the presence of IPTG. *E. coli* MG1655 WT or harbouring pNeae2 plasmid (expressing intimin Beta domain) cells were grown overnight in absence or presence of 0.25 mM IPTG to induce the production of the Intimin beta domain fused with the E-tag, Myc-tag and His-tag. Immunofluorescence have beend performed on whole cells using a rabbit anti-E-tag primary antibody and an anti-rabbit Alexa 488 conjugated secondary antibody. All images are displayed with the same constrast, scale bars = 2 µm.

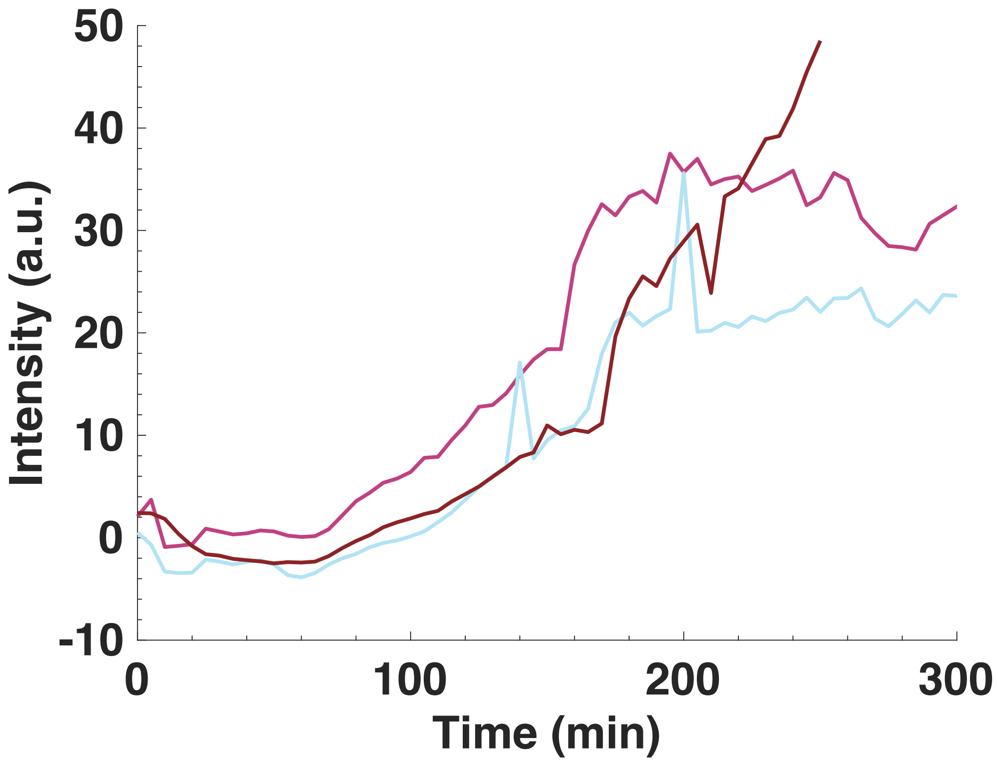

Figure S5. Dynamics of intimin-FAST in microcolonies of *E. coli*. Average intensity in microcolonies as a function of time for cells grown in LB supplemented with 40µM HBRAA-3E and IPTG at 0.5 mM for production of intimin-FAST from the plasmid pNea2-FAST. Each trace corresponds to an independent experiment.

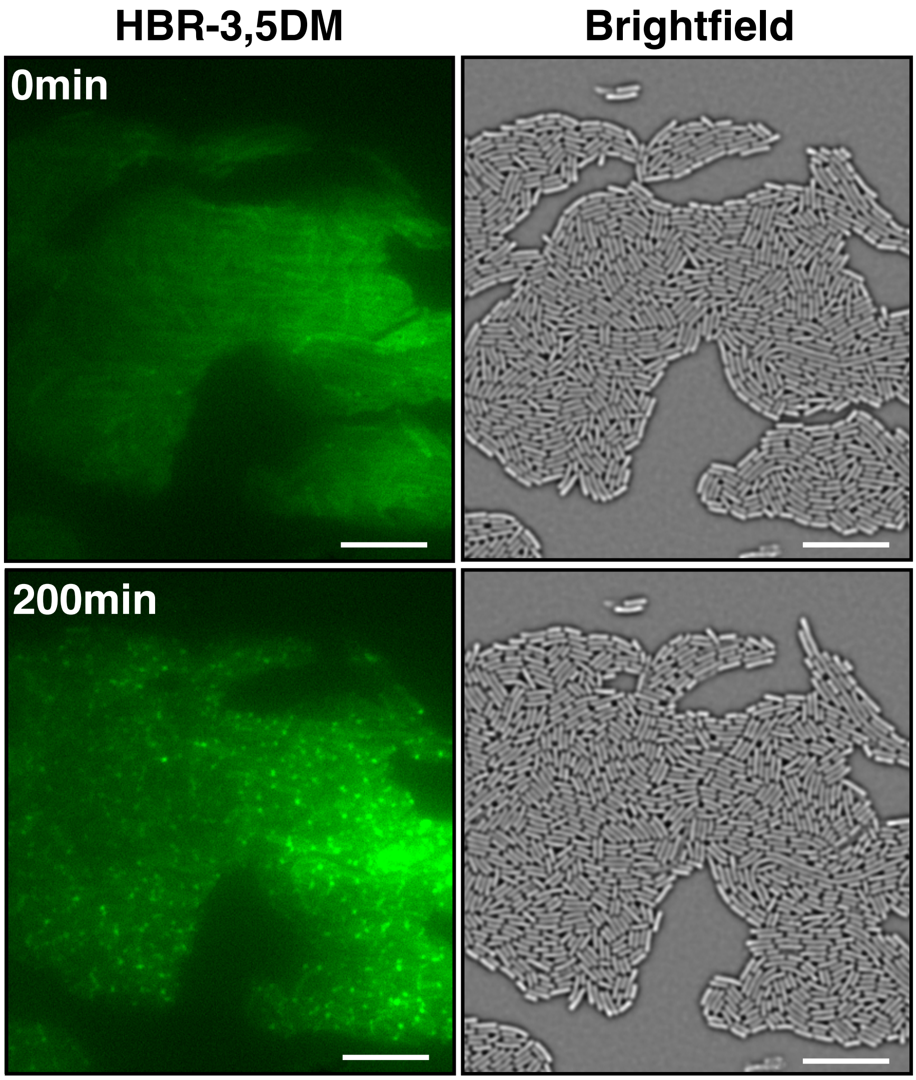

Figure S6. The intimin-FAST chimeric protein is relocalized at cell pole in stationary phase. TIRF and brightfield images of a microcolony of *E. coli* producing intimin-FAST, before (0min) and after (200min) the onset of stationary phase. The gel was loaded with 20 µM HBR-3,5DM and 0.5 mM IPTG. Scale bar = 10 µm.

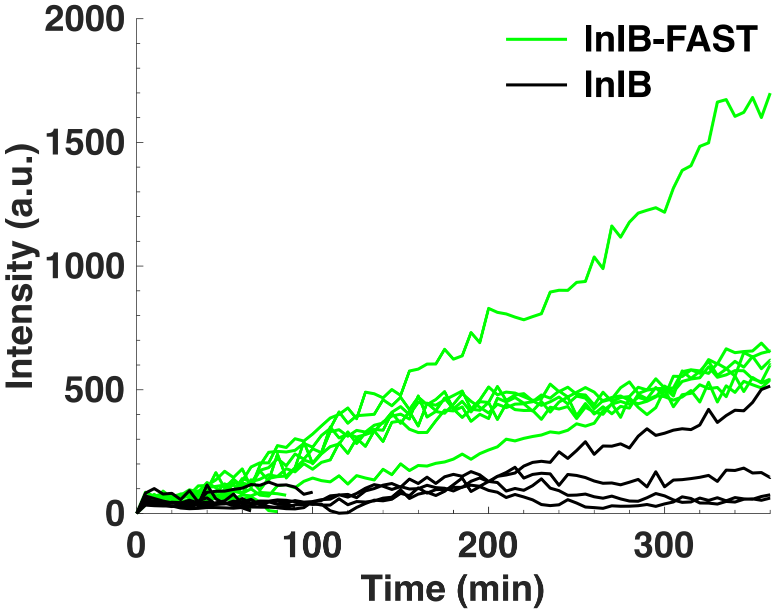

Figure S7. Dynamics of InlB-FAST in microcolonies of *L. monocytogenes*. A. Average intensity in microcolonies as a function of time for InlB-FAST (green) and InlB wild-type (black) cells growing in BHI supplemented with 20µM HBR-3,5DM. Each trace corresponds to an independent experiment.

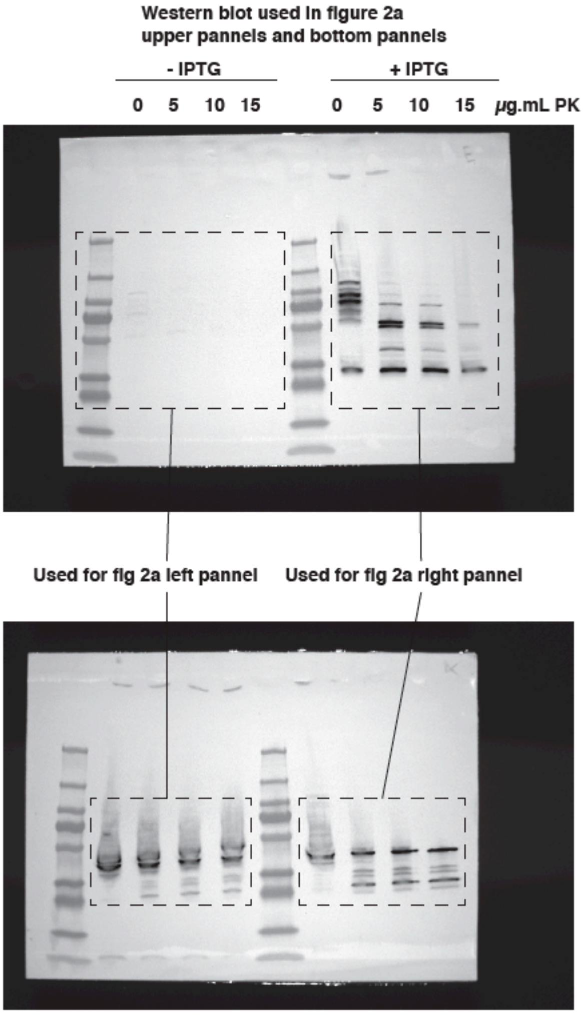

The samples have been run on two gels, transfered on two membranes and immunodetected with either anti-Etag antibodies (First western blot) or anti-RNAP alpha antibodies (second western blot).
The cropped area used to construct figure 2a, are indicated by grey dotted boxs

Western blots used for Figure 6B

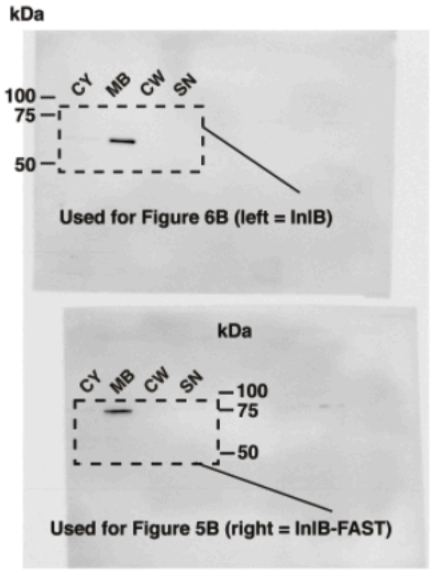

InlB blots with monoclonal antibody raised against InlB.

Fractions (CY = cytoplasm, MB = membrane, CW = cell wall, SN = supernatant) corresponding to the same culture of Listeria monocytogenes LL195 wild-type (top) and expressing inlB-FAST (bottom) have been run on two independant gels, simulteanously transferred and immunoblotted with anti-InlB antibody.

81 The cropped area used to construct Figure 6B are indicated by black dotted boxes.

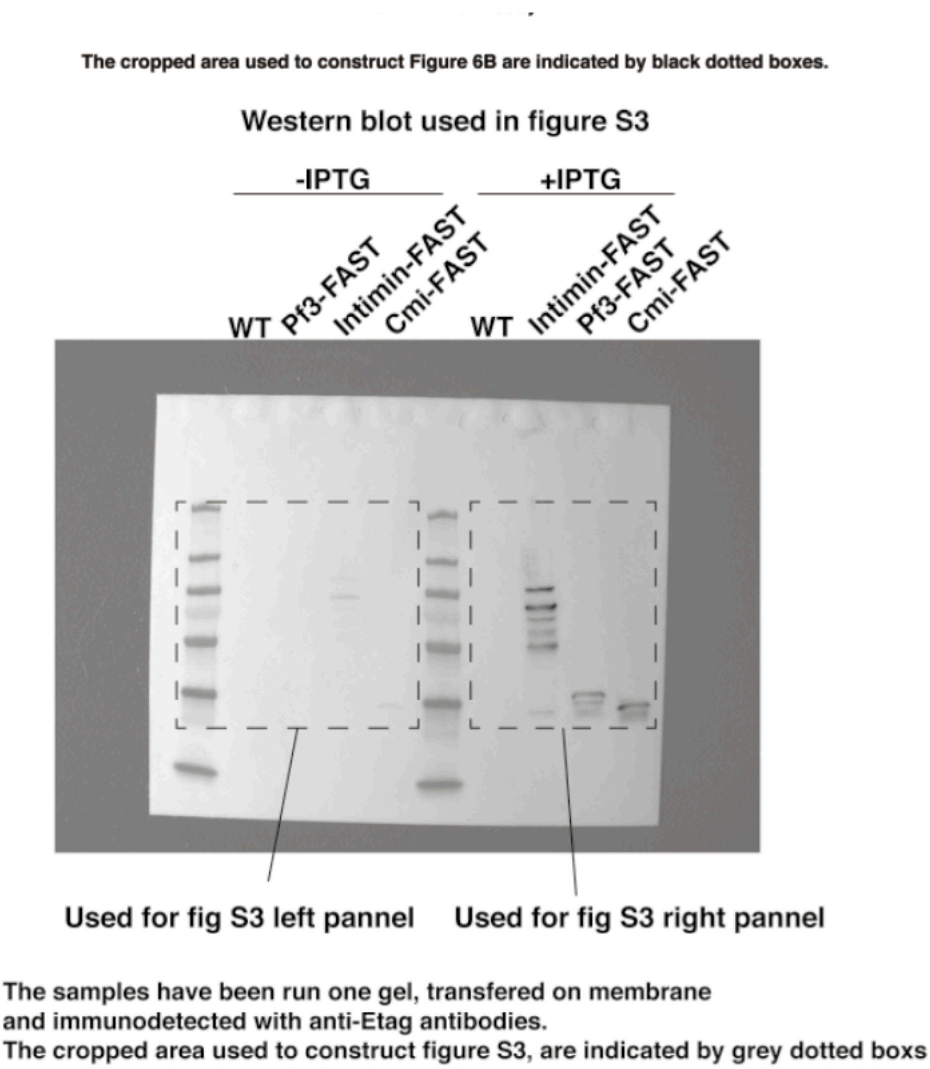

Figure S8. Full gels used in this study

<u>Supplementary Movies</u>

Movie S1: Time lapse TIRF microscopy of *E. coli* intimin-FAST microcolonies growing in LB supplemented with 40 µM HBRAA-3E. Bacteria were inoculated from a culture in stationary phase. The time between frames is 5min.

Movie S2: Time lapse brightfield microscopy of *E. coli* intimin-FAST microcolonies growing in LB supplemented with 40 µM HBRAA-3E. Bacteria were inoculated from a culture in stationary phase. The time between frames is 5min.

Movie S3: Time lapse TIRF microscopy of *E. coli* intimin-FAST microcolonies growing in LB supplemented with 20 µM HBR-3,5DM. Bacteria were inoculated from a culture in stationary phase. The time between frames is 5min.

Movie S4: Time lapse brightfield microscopy of *E. coli* intimin-FAST microcolonies growing in LB supplemented with 20 µM HBR-3,5DM. Bacteria were inoculated from a culture in stationary phase. The time between frames is 5min.

Movie S5: Time lapse TIRF and brightfield microscopy of *E. coli* intimin-FAST microcolonies growing in LB supplemented with 20 µM HBR-3,5DM and 1µg/mL mecillinam. Bacteria were inoculated from a culture in late exponential phase. The time between frames is 3min.

Movie S6: Time lapse TIRF and brightfield microscopy of *E. coli* intimin-FAST microcolonies growing in LB supplemented with 20 µM HBR-3,5DM and 100µg/mL mecillinam. Bacteria were inoculated from a culture in late exponential phase. The time between frames is 3min.

Movie S7: Time lapse TIRF and brightfield microscopy of *E. coli* intimin-FAST microcolonies growing in LB supplemented with 20 µM HBR-3,5DM and 1µg/mL A22. Bacteria were inoculated from a culture in late exponential phase. The time between frames is 3min.

Movie S8: Time lapse TIRF and brightfield microscopy of *E. coli* intimin-FAST microcolonies growing in LB supplemented with 20 µM HBR-3,5DM and 100µg/mL A22. Bacteria were inoculated from a culture in late exponential phase. The time between frames is 3min.

Movie S9: Time lapse of *L. monocytogenes* InlB-FAST microcolonies growing in BHI supplemented with 20 µM HBR-3,5DM. Brightfield and TIRF microscopy are superimposed. The contrast, of brightfield images has been inverted for clarity. Bacteria were inoculated from a culture in stationary phase. The time between frames is 5min.

Supplementary Tables

Strains	Description	Source
Escherichia coli		
MG1655	F^-,λ^-, *rph-1*	*E. coli* genetic stock center CGSC#6300
Listeria monocytogenes		
LL195	*L. monocytogenes* LL195	[38]
BIRD267	*L. monocytogenes* LL195 Δ*inlB*	This study
BIRD119	*L. monocytogenes* LL195 *inlB-FAST*	
BIRD 232	*L. monocytogenes* LL195, *prfA** *inlB-FAST*	
BIRD 234	*L. monocytogenes* LL195, *prfA**	[43]
**Plasmids**		
pNeae2	Plasmid containing N-terminal fragment of intimin with N-terminal signal peptide, periplasmic LysM domain, a β-barrel domain and the Ig-like domains (D00-D0). In the plasmid pNeae2 three different tags (*E-tag, His-tag* and myc-tag) have been fused in frame with the C-terminal end of the D0 domain. All is under control of the *pLac* promoter. CmR	[21]
pZE1R-GFP	Plasmid containing GFP under control of the phage λ *PcL* promoter. GFP is constitutively express in the cytoplasm of bacteria. AmpR	[47]
pPf3-FAST	Optimized FAST encoding gene cloned in pNeae2. Neae2 domain has been removed by PCR. FAST is fused to the Pf3 transmembrane domain. The construction is under control of the *plac* promoter. CmR	
pCmi-FAST	Optimized FAST encoding gene cloned in pNeae2. Neae2	

	domain has been removed by PCR. FAST is fused to the Cmi transmembrane domain. The construction is under control of the *plac* promoter. CmR	
pNeae2-FAST	Optimized FAST encoding gene cloned in pNeae2. FAST is fused to the β domain of the intimin. The construction is under control of the *plac* promoter. CmR	This study
pZE1R-FAST	Optimized FAST encoding gene cloned in pZE1R-GFP. GFP has been removed by PCR. FAST is under control of the phage λ *PcL* promoter and is constitutively express in the cytoplasm of bacteria. AmpR	
pMAD-*inlB*$_{1\text{-}1026}$- FAST-*inlB*$_{1027\text{-}end}$ (pBIRD111)	Plasmid for allelic replacement at the *InlB* locus: optimized *FAST* encoding gene has been inserted by PCR between the β2 and β3 leaflets of the B-repeat domain of the InlB protein (at position 1026 ± 1000 bp). ErmR (*Lm*) and AmpR (*Ec*)	
pAD-*FAST*-*Myc* (pBIRD15)	Integrative plasmid containing the optimized *FAST* encoding gene fused to a Myc-tag, under the P_{HYPER} constitutive promoter.	[43]

Table S1. Strains and plasmids used in this study

Name	Sequence
E. coli	
pZ-FAST.REV	cgtgttccatgcggtacctttctcctctttaatga
pZ-FAST.FOR	cgtgtttaataagcttaattagctgagtctagaggcat
F2_pZ_FAST.REV	agctaattaagcttattaaacacgtttaacgaaaacccagtaagagt
F2_pZ_FAST.FOR	aaggtaccgcatggaacacgttgcgttcg
F1_Neae2-FAST.FOR	aacgtgtttaataaaagcttgacctgtgaagtgaaaaat
F1_Neae2-FAST.REV	gctaccgctaccgctgccgctacctgcagctgcatcctcttctgaga
F2_Neae2-FAST.FOR	ggcagcggtagcggtagcggcagcatggaacacgttgcgttcg
F2_Neae2-FAST.REV	tcaagcttttattaaacacgtttaacgaaaacccagtaagagt
vec_Neae2_FAST_CMI.F	aaggcgaatttaacaacaacggtgcgccggtgc
vec_Neae2_FAST_CMI.R	ttcatgctaatcactttcatgatttgccctcgttatctagaaat
CMIforNeae2_FAST_CMI.F	ctagataacgagggcaaatcatgaaagtgattagcatgaaatttatttttattctgacca
CMIforNeae2_FAST_CMI.R	ggatacggcaccggcgcaccgttgttgttaaattcgcctttatcttcgc
Vec_Neae2_FAST_pf3.F	tctttgaatttaacaacaacggtgcgccggtgc
Vec_Neae2_FAST_pf3.R	tcagtaatcacggattgcatgatttgccctcgttatctagaaat
pf3for_Neae2_FAST_pf3.R	ggatacggcaccggcgcaccgttgttgttaaattcaaagaattgcgctttg
pf3for_Neae2_FAST_pf3.F	ctagataacgagggcaaatcatgcaatccgtgattactgatgtgac
L . monocytogenes	
oAL833 (3'-*inlB* LL195 (-1000 from position 1026) – BglII-pMAD, Fw)	gacagatctgaaggaaatattgtttaatctcagg
oAL834 (*inlB* LL195(to position 1026)-GGSG linker -3'-*FAST*, Rv)	catgccgctcgagcccgtccctgcctctacttttg
oAL835 (*inlB* LL195(to position 1026)-GGSG linker-3'-*FAST*, Fw)	gacgggctcgagcggcatggaacatgttgctttcggttc
oAL836 (5'-*FAST*-AAAG linker-*inlB* from position 1027, Rv)	tcggcccgcggccgctacacgtttaacaaaaaccc
oAL837 (5'-*FAST*-AAAG linker-*inlB* from position 1027, Fw)	gtagcggccgcgggccgaataactgcacctaaacc
oAL838 (5'-*inlB* LL195(+1000 from position 1026)-SalI-pMAD, Rv)	gacgtcgacccggccgcagaatatgg

Table S2. Primers used in this study

References

21 Salema, V. *et al.* Selection of single domain antibodies from immune libraries displayed on the surface of E. coli cells with two β-domains of opposite topologies. *PLoS One* **8**, e75126-e75126, https ://doi:10.1371/journal.pone.0075126 (2013).

38 Toledo-Arana, A. *et al.* The Listeria transcriptional landscape from saprophytism to virulence. *Nature* **459**, 950-956, https ://doi:10.1038/nature08080 (2009).

43 Peron Cane, C. *et al.*, Fluorescent secreted bacterial effectors reveal an intravacuolar replication compartment for Listeria monocytogenes. BioRxiv https ://doi.org/10.1101/2019.12.23.88668 9 (2020).

47 Lutz, R. & Bujard, H. Independent and tight regulation of transcriptional units in Escherichia coli via the LacR/O, the TetR/O and AraC/I1-I2 regulatory elements. *Nucleic acids research* **25**, 1203-1210, https ://doi:10.1093/nar/25.6.1203 (1997).
